# Genome-wide screening of mouse knockouts reveals novel genes required for normal integumentary and oculocutaneous structure and function

**DOI:** 10.1038/s41598-019-47286-2

**Published:** 2019-08-01

**Authors:** Bret A. Moore, Ann M. Flenniken, Dave Clary, Ata S. Moshiri, Lauryl M. J. Nutter, Zorana Berberovic, Celeste Owen, Susan Newbigging, Hibret Adissu, Mohammad Eskandarian, Colin McKerlie, International Mouse Phenotyping Consortium, Steve Brown, Sara Wells, Ann-Marie Mallon, Arthur L. Beaudet, Martin Hrabe de Angelis, Natasha Karp, Bob Braun, Yann Herault, Xiang Gao, Yuichi Obata, Paul Flicek, Terrence Meehan, Helen Parkinson, Damian Smedley, J. K. Seong, Glauco Tocchini-Valentini, Fabio Mammano, Sara M. Thomasy, K. C. Kent Lloyd, Christopher J. Murphy, Ala Moshiri

**Affiliations:** 1 William R. Pritchard Veterinary Medical Teaching Hospital, School of Veterinary Medicine, University of California Davis, Davis, CA United States; 2 The Centre for Phenogenomics, Toronto, ON M5T 3H7 Canada; 3 Lunenfeld-Tanenbaum Research Institute, Mount Sinai Hospital, Toronto, ON M5G 1X5 Canada; 4 Department of Surgery, School of Medicine, and Mouse Biology Program, University of California Davis, Davis, CA United States; 5 Division of Dermatology, Department of Medicine, University of Washington, Seattle, WA United States; 6 The Hospital for Sick Children, Toronto, ON M5G 1X8 Canada; 7 Department of Surgical and Radiological Sciences, School of Veterinary Medicine, University of California Davis, Davis, CA United States; 8 Department of Ophthalmology & Vision Science, School of Medicine, University of California Davis, Sacramento, CA United States; 9 Medical Research Council Harwell Institute (Mammalian Genetics Unit and Mary Lyon Centre), Harwell, Oxfordshire OX11 0RD UK; 10 Department of Molecular and Human Genetics, Baylor College of Medicine, Houston, TX 77030 USA; 11 German Mouse Clinic, Institute of Experimental Genetics, Helmholtz Zentrum München, German Research Center for Environmental Health, Ingolstädter Landstraße 1, 85764 Neuherberg, Germany; 12 The Wellcome Trust Sanger Institute, Wellcome Genome Campus, Hinxton, Cambridge CB10 1SA UK; 13 The Jackson Laboratory, Bar Harbor, ME 04609 USA; 14 Institut de Génétique et de Biologie Moléculaire et Cellulaire, Université de Strasbourg, 1 rue Laurent Fries, 67404 Illkirch, France; 15 Centre National de la Recherche Scientifique, UMR7104 Illkirch, France; 16 Institut National de la Santé et de la Recherche Médicale, U1258 Illkirch, France; 17 Université de Strasbourg, 1 rue Laurent Fries, 67404 Illkirch, France; 18 CELPHEDIA, PHENOMIN, Institut Clinique de la Souris (ICS), CNRS, INSERM, University of Strasbourg, 1 rue Laurent Fries, 67404 Illkirch, Graffenstaden France; 19 SKL of Pharmaceutical Biotechnology and Model Animal Research Center, Collaborative Innovation Center for Genetics and Development, Nanjing Biomedical Research Institute, Nanjing University, Nanjing, 210061 China; 20 RIKEN BioResource Center, Tsukuba, Ibaraki 305-0074 Japan; 21 European Molecular Biology Laboratory, European Bioinformatics Institute, Wellcome Genome Campus, Hinxton, Cambridge CB10 1SD UK; 22 Clinical Pharmacology, Charterhouse Square, Barts and the London School of Medicine and Dentistry, Queen Mary University of London, London, EC1M 6BQ UK; 23 Korea Mouse Phenotyping Consortium (KMPC) and BK21 Program for Veterinary Science, Research Institute for Veterinary Science, College of Veterinary Medicine, Seoul National University, 599 Gwanangno, Gwanak-gu, Seoul 08826 South Korea; 24 Monterotondo Mouse Clinic, Italian National Research Council (CNR), Institute of Cell Biology and Neurobiology, Adriano Buzzati-Traverso Campus, Via Ramarini, I-00015 Monterotondo Scalo, Italy

**Keywords:** Eye diseases, Skin diseases, Genetics research

## Abstract

Oculocutaneous syndromes are often due to mutations in single genes. In some cases, mouse models for these diseases exist in spontaneously occurring mutations, or in mice resulting from forward mutatagenesis screens. Here we present novel genes that may be causative for oculocutaneous disease in humans, discovered as part of a genome-wide screen of knockout-mice in a targeted single-gene deletion project. The International Mouse Phenotyping Consortium (IMPC) database (data release 10.0) was interrogated for all mouse strains with integument abnormalities, which were then cross-referenced individually to identify knockouts with concomitant ocular abnormalities attributed to the same targeted gene deletion. The search yielded 307 knockout strains from unique genes with integument abnormalities, 226 of which have not been previously associated with oculocutaneous conditions. Of the 307 knockout strains with integument abnormalities, 52 were determined to have ocular changes attributed to the targeted deletion, 35 of which represent novel oculocutaneous genes. Some examples of various integument abnormalities are shown, as well as two examples of knockout strains with oculocutaneous phenotypes. Each of the novel genes provided here are potentially relevant to the pathophysiology of human integumentary, or oculocutaneous conditions, such as albinism, phakomatoses, or other multi-system syndromes. The novel genes reported here may implicate molecular pathways relevant to these human diseases and may contribute to the discovery of novel therapeutic targets.

## Introduction

Phakomatoses traditionally comprise the group of genetic disorders with structural abnormalities found in tissues that arise from embryonic ectoderm, namely the central nervous system, eyes and skin, and are thus dubbed neuro-oculo-cutaneous syndromes^1^. Most of these entities are single gene disorders. Examples of human phakomatoses with heritable mutations include, tuberous sclerosis (autosomal dominant mutations in *TSC1* or *TSC2*), neurofibromatosis (autosomal dominant mutations in *NF1* or *NF2*), von Hippel-Lindau disease (autosomal dominant mutations in *VHL*), basal cell nevus syndrome (autosomal dominant mutations in *PTCH*), incontinentia pigmenti (*X-*linked dominant mutations in *NEMO*), and ataxia telangiectasia (autosomal recessive mutations in *ATM*). Some phakomatoses, such as Sturge-Weber syndrome (a nonhereditary congenital disorder resulting from a somatic activating mutation in *GNAQ*) and Wyburn-Mason syndrome (a nonhereditary congenital and sporadic condition) occur with no known pattern of inheritance or incidence. Yet other syndromes affecting these same organ systems but with abnormalities derived from mesenchymal or endodermal (rather than ectodermal) tissues are thought by some to be included in the phakomatoses. An example of such a disease is PHACES (posterior fossa malformations, hemangiomas, arterial anomalies, cardiac anomalies or aortic coarctation, eye abnormalities, sternal clefting or supraumbilical raphe), a congenital syndrome with similar incidence to Sturge-Weber that currently has no known cause.

Similar to phakomatoses, albinism refers to a set of genetic disorders caused by autosomal recessive mutations in genes relevant to pigmentation, often resulting in abnormalities either in the eyes alone (ocular albinism, OA), or the eyes and integument (oculocutaneous albinism, OCA)^2^. To date, only one locus has been associated with OA (mutations in *GPR143*); by contrast, seven types of OCA have been described with mutations in six genes identified to date (*TYR*, *OCA2*, *TYRP1*, *SLC45A2*, *SLC24A5*, *C10orf11*). OCA has also been observed as part of several broader syndromes, including Hermansky-Pudlak (*HPS1*, *AP3B1*, *HPS3*, *HPS4*, *HPS5*, *HPS6*, *DTNBP1*, *BLOC1S3*, and *BLOC1S6* genes), Chediak-Higashi (*LYST*), and Griscelli (*MYO5A*, *RAB27A*, and *MLPH*) syndromes. Despite recent advances in sequencing, approximately 20 percent of patients with OA and/or OCA still have no identified mutation accounting for their phenotype.

Given the ample number of known single gene disorders that have overlapping integument and ocular involvement, as well as the number of oculocutaneous syndromes with possible genetic mutations that have not yet been identified, we sought to query the International Mouse Phenotyping Consortium (IMPC) database for novel single-gene targeted loss of function (knockout) mouse strains with integument and ocular abnormalities. The IMPC was established in 2011 as a coordinated program of highly specialized academic health sciences centers with expertise in high-throughput mouse mutagenesis and/or comprehensive phenotyping^3^. The goal of the IMPC is to create the first functional catalogue of the mammalian genome, as represented by the laboratory mouse^4,5^. Now encompassing 19 centers in 15 countries on 5 continents, the IMPC has, as of April 3, 2018, produced fully validated knockout mouse strains for 6,000 genes and completed phenotyping across 11 body systems for over 5,000 of these genes. Phenotyping was conducted on cohorts of 7 male and 7 female mutant mice on a C57BL/6N genetic background beginning at 4 weeks of age with weekly body weights, sequential non-invasive or minimally invasive phenotyping tests, and a panel of terminal tests at 16 weeks of age. Testing and data collection at all centers was done using IMPC standardized phenotyping protocols (http://www.mousephenotype.org/impress), that enable uniform principles of scientific rigor and reproducibility^6^. Approximately 30% of strains were homozygous lethal^7^. All data and images captured and curated by the phenotyping centers are uploaded to the IMPC Data Coordination Center (DCC), quality-controlled, and analyzed using a robust statistical analysis pipeline^8^, then posted to the IMPC portal (www.impc.org). All of the raw and analyzed data are viewable online and accessible for download and manipulation.

We report here the genes that are likely causative for oculocutaneous disease in mice, each of which are potentially relevant to the pathophysiology of human OA, OCA, and/or phakomatoses. Any genetic underpinnings of these diseases are of interest not only for further understanding of their etiologies, but also for identification of potential targets for gene or drug therapy.

## Results

### Integumentary data base search

All wild-type mice produced on the C57BL/6N background had a uniform black coat colour. Searching the IMPC database for knockout strains with integument defects identified 307 knockout strains (Supplementary Table 1). Phenotype details of the abnormalities are reported in the phenotype descriptions available on the IMPC web portal for some strains. Among the 307 knockout strains identified as having an integument phenotype, 81 have a previously described role in cutaneous biology (*Abcb6*, *Abcd4*, *Acer1*, *Actg1*, *Acvr1b*, *Acvr2b*, *Aldh2*, *Alg1*, *Alg8*, *Ankle1*, *Arpc2*, *Arpc4*, *Ash1l*, *B2m*, *Barx2*, *Bms1*, *Cadm1*, *Carmil2*, *Cast*, *Cd109*, *Cd248*, *Cd5l*, *Cdc7*, *Chst8*, *Cog6*, *Cxcl17*, *Dbn1*, *Dct*, *Def6*, *Dgat2l6*, *Dnase1l2*, *Dnm1l*, *Dsg1b*, *Dtnbp1*, *Eif2s2*, *Far2*, *Foxn1*, *Furin*, *Gdap1*, *Gpr65*, *Gsta4*, *Igfbp3*, *Il33*, *Ing4*, *Kdm4c*, *Krt31*, *Krtap17-1*, *Lama4*, *Lor*, *Lrig1*, *Lst1*, *Lyst*, *Mc1r*, *Mcub*, *Mplkip*, *Mpz*, *Myo10*, *Myo5a*, *Mysm1*, *Nacc1*, *Nampt*, *Nfkb1*, *Ovol1*, *P3h1*, *Per2*, *Ppl*, *Prss53*, *Ptprd*, *Rad18*, *Rassf8*, *Reql4*, *Ripk3*, *Rspo1*, *Sema3f*, *Slc17a9*, *Slc24a5*, *Smc3*, *Sparc*, *Svep1*, *Tmem79*, *and Ywhaz*). A total of 58 of these 81 genes have published knockout mouse models, while a knockout strain for the remaining genes with a cutaneous phenotype have not been reported. A total of 134 of the 307 genes have published knockout mouse models, 76 of which have no reported integument phenotype. For example, one of the genes that does not have a published knockout mouse strain, *structural maintenance of chromosomes 3* (*Smc3*), is known to be associated with hair abnormalities as part of the constellation of abnormalities seen in Cornelia de Lange syndrome in humans, characterized by bushy eyebrows and long curly eyelashes^9^. In total, 226 of the genes reported here have no published integument phenotype information and therefore represent novel integument disease genes.

### Oculocutaneous data base search

To determine if any of the knockout strains with integument phenotypes also had ocular phenotypes, the 307 genes with cutaneous and/or hair coat colour phenotypes were searched in the IMPC database for coexisting ocular abnormalities detected during the eye test of the IMPC phenotyping pipeline. A total of 52 of these knockout strains were found to have concomitant ocular phenotypes (Supplementary Table 2). Seventeen of these 52 genes have previously described ocular and integumentary associations (e.g. *Sparc*^10,11^ and *Slc24a5*^12–14^, and 22 genes have a previously described knockout mouse model (e.g. *Barx2*^15,16^. In total, 35 genes with oculocutaneous phenotypes were novel findings (*Acvr2b*, *Alg10b*, *Anapc15*, *Ash1l*, *Bms1*, *Carmil2*, *Chic2*, *Chst8*, *Clk1*, *Cog6*, *Cotl1*, *Cox6b1*, *Dbn1*, *Dcp2*, *Dnase1l2*, *Dsg1b*, *Ggps1*, *Gp6*, *Hdgfl3*, *Kdm8*, *Klhdc2*, *Mogs*, *Mysm1*, *Nadk2*, *Nfkb1*, *Plac8*, *Rcc2*, *Sdr42e1*, *Sgip1*, *Sik3*, *Slc17a9*, *Slc38a10*, *Tcf7*, *Tmem30b*, *Tuft1*). Several genes have been widely reported to be involved with normal function of both integumentary and ocular tissues. Other genes have described integument involvement but no reported ocular function (e.g. *Nfkb1*, *Mysm1*, *Dsg1b*, *Dnase1l2*, *Dbn1*, *Cog6*, *Chst8*, *Carmil2*, *Bms1*, *Ash1l*, *Acvr2b*).

## Discussion

### Integument phenotypes

**Figure 1:**
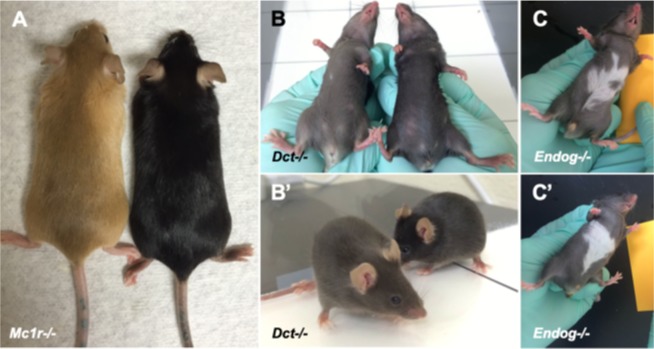
Examples of various types of coat color abnormalities seen in knockout mice. *Mc1r* knockout mice (**A**, left) have uniformly hypopigmented strawberry blonde coat color compared with wild type C57BL/6N (**A**, right) control mice. Mice with targeted deletion of the *Dct* gene have a brown-colored coat (**B**,**B’**, left) compared with wild type C57BL/6N (**B**,**B**’, right) control mice. Knockout mice with a white patch on the abdomen have been observed with variable penetrance. Just three of eight *Endog* knockout mice were found to have a white patch on the belly (**C**,**C’**), which may be sporadic, or may represent reduced penetrance related to the targeted deletion.

Among the 307 mouse strains that were found to have integument phenotypes, several coat pigmentation abnormalities were observed. Homogenous alteration in coat coloration was noted in some cases, such as in homozygous null *Mc1r*^*tm1*.*1*(*KOMP*)*Vlcg*^ and *Dct*^*tm1b*(*KOMP*)*Mbp*^ mice (hereafter defined as *Mc1r*^*−/−*^ and *Dct*^*−/−*^ respectively) (Fig. 1A,B). *Mc1r*^*−/−*^ mice (Fig. 1A, left) have a uniformly hypopigmented coat color of strawberry blonde compared to uniformly black hair coat color of wild-type C57BL/6N controls (Fig. 1A, right). *Dct*^*−/−*^ mice have a gray coat color (Fig. 1B,B’, left) compared to C57BL/6N controls (Fig. 1B,B’, right). Heterogenous coat color changes were also noted, such as a white patch of hair coat on the abdomen. An example, homozygmous null *Endog*^*tm1*.*1*(*KOMP*)*Vlcg*^ mice (hereafter defined at *Endog*^*−/−*^), were observed and annotated to have a white patch on the ventral abdomen (Fig. 1C,C’). However, only 3 out of 8 *Endog*^*−/−*^ had a white patch, which may either represent reduced penetrance of the *Endog*^*−/−*^ phenotype or a nonspecific phenotype unrelated to this gene. A total of 76 of the 307 knockout mouse strains reported here have previously described knockout mouse models but no integument phenotypes were reported. Allelic differences in the approach of the knockout strategy, strain-specific differences, and possible lack of careful attention to integument phenotypes may in part explain these discrepancies.

### Oculocutaneous phenotypes

**Figure 2:**
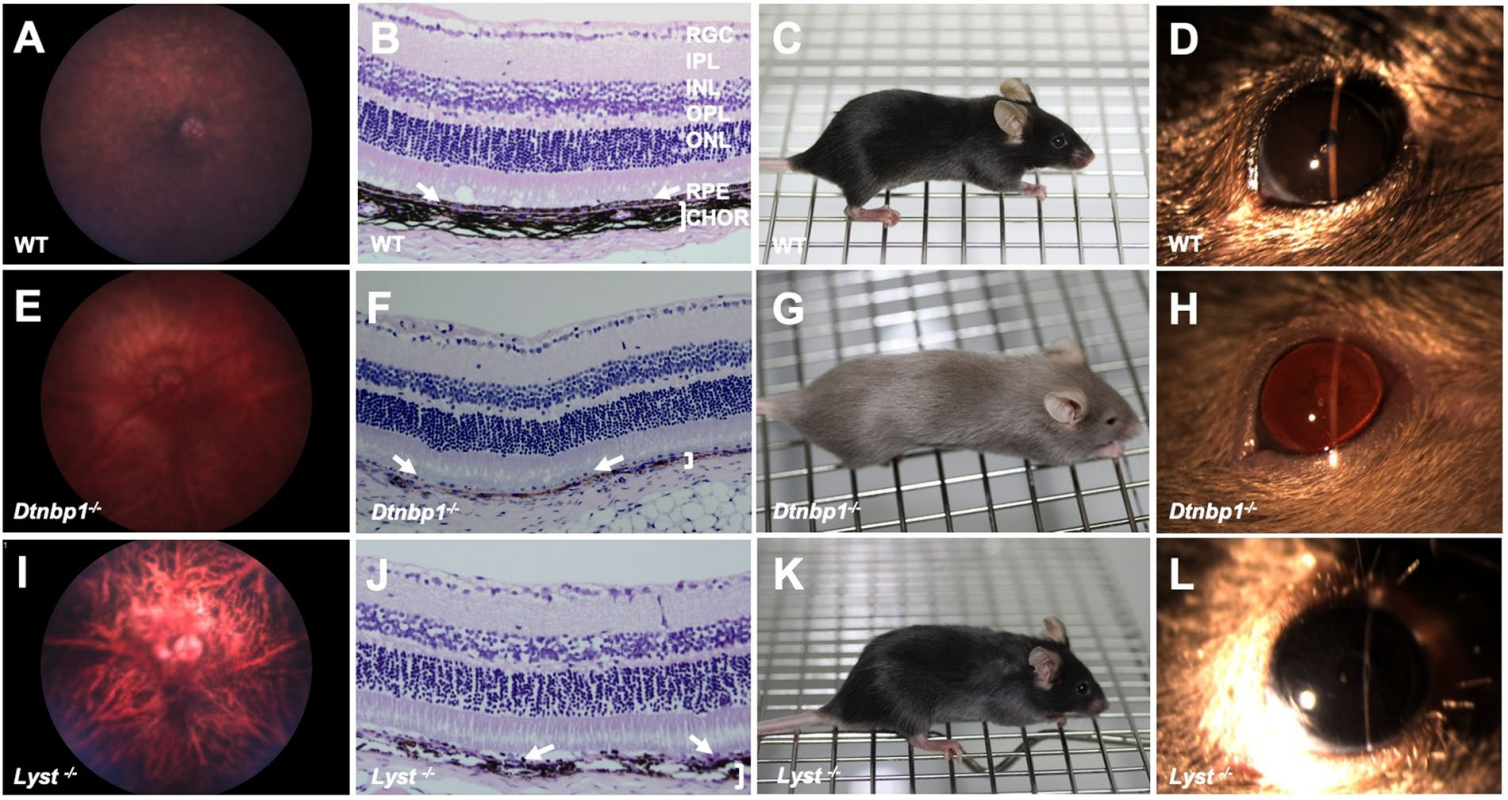
Mouse models of syndromic oculocutaneous albinism. Wild type control C57BL/6N mouse with normal retinal pigmentation (**A**), normal pigmentation of the retinal pigmented epithelium (arrows) and choroid (bracket) (**B**), normal black coat and cutaneous pigmentation (**C**), and normal iris pigmentation (**D**). In contrast, *Dtnbp1*^*−/−*^ mice, which recapitulate features of Hermansky-Pudlak syndrome, have severe hypopigmentation of the ocular fundus (**E**), marked RPE (arrows) and choroidal hypopigmentation (bracket) seen on histology (**F**), marked cutaneous and hair hypopigmentation (**G**), and transillumination defects of the iris due to severely reduced pigmentation (**H**). Mice with targeted deletion of *Lyst*, which recapitulate features of Chediak-Higashi syndrome, have moderately reduced fundus pigmentation (**I**), moderately reduced pigmentation of the RPE (arrows) and choroid (bracket) on histology (**J**), gray coat color (**K**), and a gray iris appearance (**L**). RGC (retinal ganglion cell layer), IPL (inner plexiform layer), INL (inner nuclear layer), IPL (inner plexiform layer), ONL (outer nuclear layer), RPE (retinal pigmented epithelium layer, arrows pointing to this monolayer of cells in **B**,**F**,**J**), CHOR (choroidal layer, demarcated by bracket in **B**,**F**,**J**).

Of the 52 genes found to have both integument and eye phenotypes, 3 had ocular pigmentation abnormalities. Wild-type C57BL/6N controls had normal ocular fundus appearance (Fig. 2A), darkly pigmented choroidal melanocytes and retinal pigmented epithelium (RPE) (Fig. 2B), expected cutaneous pigmentation and black hair coat (Fig. 2C), and brown irides (Fig. 2D). Homozygous null *Dtnbp1*^*tm1a*(*EUCOMM*)*Hmgu*^ (hereafter *Dtnbp1*^*−/−*^) mice have a lightly pigmented fundus with clearly visible choroidal vasculature (Fig. 2E), RPE and choroidal melanocyte hypopigmentation detectable in histological section (Fig. 2F), hypopigmentation of the skin and hair (Fig. 2G), and transillumination defects of the iris due to reduced pigmentation (Fig. 2H). These findings are consistent with human patients with mutations in *DTNBP1*, who are affected with cutaneous albinism, iris and retinal ocular albinism on eye exam, and RPE hypopigmentation apparent on histology^17^. The targeted deletion of the *Dtnbp1* gene produced by the IMPC recapitulates the main features of Hermansky-Pudlak syndrome (HPS), a rare autosomal recessive genetic disorder affecting roughly 1/500,000 individuals worldwide, but disproportionately present in 1/1800 people in Puerto Rico^18,19^. The disease causes a constellation of oculocutaneous albinism, in addition to platelet abnormalities, and accumulation of ceroid lipofuscin^20^. The pathophysiological basis of Hermansky-Pudlack syndrome is thought to be secondary to lysosomal dysfunction^21^. Mutations in at least nine different genes can cause this disorder, including *HPS1*, *HPS2* (aka *AP3B1*), *HPS3*, *HPS4*, *HPS5*, *HPS6*, *HPS7* (aka *DTNBP1*), *BLOC1S3*, and *BLOC1S6*^2,22^. Diagnosis of HPS is based on the presence of hypopigmentation of the skin, hair, and eyes, as well as decreased dense bodies in platelets. Molecular testing is commercially available for *HPS1*, *HPS3*, and *HPS4*.

*Dtnbp1*^*−/−*^ produced by the IMPC is also similar to the phenotype of the spontaneously-occurring *Sdy* mouse^18^. *Sdy* mice have a large (~38 kb) deletion eliminating exons 6 and 7 of the *Dtnbp1* gene, which codes for Dysbindin protein^23^. Some human patients with HPS harbor mutations in this gene, and Dysbindin is a component of the biogenesis of lysosome-related organelles complex 1 (BLOC-1). The BLOC-1 complex includes the proteins Pallidin, Muted, and Cappuccino, which are associated with HPS in mice. These proteins act in a coordinated fashion to regulate trafficking to lysosome associated organelles. Melanosomes are one such organelle, depending on BLOC-1 function. Both RPE cells and choroidal melanocytes in *Sdy* mouse eyes have pigmented granules that are abnormal in appearance and fewer in number compared to wild-type controls^23^. The findings reported here strongly resemble the previously described phenotype of the spontaneous mutation of *Dtnbp1* in *Sdy* mice, and also supports the role of dysbindin protein in HPS.

The beige mutant mouse strain, originally reported in 1967, has a lighter coat color than wild-type mice^24^. The mutation associated with this strain’s coat color phenotype is located in the *Lyst* gene. *Lyst* mutations are associated with Chediak-Higashi syndrome (CHS) in humans, an oculocutaneous albinism syndrome analogous to HPS. The IMPC has produced a targeted homozygous null *Lyst* mutation (*Lyst*^*tm1b*(*EUCOMM*)*Wtsi*^, hereafter *Lyst*^*−/−*^). *Lyst*^*−/−*^ mice have reduced pigmentation of the RPE and thus highly visible underlying choroidal vasculature (Fig. 2I). The choroidal melanocytes beneath the RPE appear to be moderately hypopigmented (Fig. 2J) compared to C57BL/6N wild-type control animals (Fig. 2B). The hair coat color is slightly less pigmented resulting in a gray color (Fig. 2K) compared to wild-type C57BL/6N littermates (Fig. 2C). The iris is dark gray in *Lyst*^*−/−*^ mice, with no other obvious iris abnormalities noted (Fig. 2L).

Chediak-Higashi syndrome is a very rare autosomal recessive disease with less than 500 cases reported in the literature^25^. The clinical syndrome consists of oculocutaneous albinism, in addition to immunodeficiency, and neuropathy, and is considered primarily to be a lysosomal disorder^26,27^. It has been described in many mammalian species, including rats^28^, cows^29–31^, cats^32^, mink^29^, foxes^33,34^, and orca^35^. Spontaneous mutations in mice, which recapitulate the main features of CHS were originally published more than 60 years ago^36–38^. The coat color of these mice is hypopigmented, so the name “beige” was adopted to describe their phenotype^39^. “Beige” mice have reduced pigmentation in hair, skin, and eyes^40^. A second “beige” mutation occurred on a non-agouti background, and these animals have a charcoal gray coat color. The coat color is dependent on the genetic background of the animal^41^. “Beige” mice have pigment granules, but they are much larger and fewer in number than in control mice, and they are clumped together, giving the animals an overall hypopigmented appearance. Similarly, white blood cells and various cells of the lungs and kidneys also have giant granules^24,41^. A progressive, insidious neurological disease affects the mice as they age^41^. Immunodeficiency has also been described in “beige” mice, specifically altering natural killer cell function, T-cell response to tumors, and decreased ability to kill bacteria, making these mice susceptible to infection^42,43^. “Beige” mice weigh less and die younger than controls^41^.

Genetic analysis of a spontaneous mutation of “beige” mice revealed that the “beige” allele causes a deletion of a single amino acid (isoleucine) from the WD40 domain of the Lyst (lysosomal trafficking regulator) protein^44^. Another spontaneous *Lyst* deletion was the result of an insertion of a LINE-1 element resulting in expression of a predicted truncated protein lacking the C-terminus, including the WD40 domain^45,46^. The location within the WD40 domain suggests that the mutation may disrupt a protein-protein interaction^47^. A total of 9 spontaneously occuring *Lyst* mutations are reported in the MGI mouse mutant index (data release 7.0).

CHS in humans is caused by mutations in the *CHS1*^48^. Runkel *et al*. reported another mutation in the *Lyst* gene which they discovered in an ENU mutagenesis screen, resulting in a mouse with gray coat color which they named *Lyst*(bg-gray)^49^. Similar to studies in “beige” mice, the authors reported enlarged and irregular melanosomes in all pigmented cell types. The knockout mutation produced by the IMPC and reported here was made on the C57BL/6N background strain. This mutant strain has a gray rather than beige coat color due to the dark coat pigmentation of the background strain. The cutaneous and ocular pigmentation abnormalities identified in *Lyst*^*−/−*^ mice by the IMPC are similar to those reported previously^50–55^. The *Lyst*^*−/−*^ mice in this report have gray coat color but no detectable iris pigmentation abnormalities (Fig. 2L), in agreement with a previous report, that demonstrated only very subtle iris transillumination defects^44^. Ultrastructural analysis of the iris has shown enlarged melanosomes in beige mice^44^. The fundus images of IMPC *Lyst*^*−/−*^ mice showed a reduced pigmentation pattern (Fig. 2I) that makes the choroidal vasculature more apparent compared to wild-type C57BL/6N control fundus (Fig. 2A). This distinctive pattern of choroidal vasculature can be described as tigroid, and is a feature of albinism^56^ and some other pathological retinal states in human patients^57^.

### Novel oculocutaneous genes

The advantages of searching the IMPC data base to identify candidate genes for human syndromes are that mammalian genes can be targeted in mice but not in humans. Furthermore, the IMPC aims to knockout all genes in the mouse genome making it an unbiased approach to discover novel disease genes. This approach is particularly valuable for discovering genes relevant to multisystemic human syndromes since the phenotyping process in knockout mice spans all organ systems and is harmonized between IMPC centers. Although there are distinct biological similarities between mouse and human eyes, the obvious differences between human and mouse are potential disadvantages, and disease genes in mice may not correlate to similar pathologies in humans in every case. The fact that a significant number of disease genes discovered through this study agrees with previously published phenotypes in mice and human patients validates the overall approach of the IMPC program in general. It is important to emphasize that only statistically significant phenotypes are reported by the IMPC, and partially penetrant phenotypes not meeting statistical significance such as the hypopigmented belly patch in the *Endog*^*−/−*^ mice may be sporadic and unrelated to the function of this gene.

We report here 226 novel genes associated with integument phenotypes using the power of reverse genetic screening in mouse strains with knockout mutations. Among these 226 novel cutaneous genes, 35 also had novel ocular phenotypes. These genes may eventually prove to be relevant in human phakomatoses, albinism, or other syndromes. If these genes are relevant to human patient populations, then these knockout strains may be useful animal models for testing pharmacologic, genetic, or cell-based therapies. All of the IMPC knockout strains reported here are readily available to the scientific community from IMPC repositories as frozen germplasm (e.g. www.mmrrc.org). Furthermore, the phenotype findings reported here in mice may help clinicians identify a more complete clinical picture of human patients found to have disease-causing mutations in these genes. Lastly, the molecular pathways in which these targeted genes function may shed light onto the mechanisms underlying dermatologic and oculocutaneous diseases.

## Materials and Methods

### Animals

Strict ethical review licensing and accrediting bodies were used by all IMPC centers that are reflective of their national legislation (Institutional Animal Care and Usage Committees, Regierung von Oberbayern, Com’Eth, Animal Welfare and Ethical Review Bodies, RIKEN Tsukuba Animal Experiments Committee, and Animal Care Committee). Phenotyping procedures were routinely assessed for animal welfare to minimize suffering.

An initial list of integument phenotypes was developed by searching the IMPC site under the ‘phenotypes’ tab for all integument phenotypes. This enabled the widest possible net to be populated, encompassing non-specific integument abnormalities (e.g. hair, skin, nails, etc.). A complete gene list from each phenotype was then exported to a spreadsheet, and a total gene population with all associated integument phenotypes was curated. The IMPC dataset returned a list of 307 genes associated with cutaneous phenotypes. These 307 genes were then cross-referenced with phenotypes related to ocular (eye) morphology, as well as other systemic phenotypes, by searching each gene individually. A total of 52 genes were identified to have both integument *and* ocular phenotypes. All genes with an integument phenotype were then queried in a literature search, specifically by searching www.pubmed.gov and www.google.com/scholar for each gene and the search term “skin,” “hair,” and “integument,” and similarly for the oculocutaneous genes for “eye” and/or the anatomical structure within the eye affected by the reported phenotype, such as “retina”. Each gene was then categorized as having no known association with a mouse integument or ocular abnormality, or a human integument or ocular abnormality. An additional search was then completed for each gene using the search term “knockout” to evaluate if a mouse knockout model has been produced for any of the identified genes outside of the IMPC.

### Ophthalmic phenotyping

Complete ophthalmic examinations were done on both eyes of each mouse at 15–16 weeks of age. A standardized phenotyping protocol for evaluation of ocular and adnexal structures was followed by all phenotyping centers. Examinations were carried out by highly trained and experienced technical staff using ocular imaging equipment that was overseen by lead site scientists. Examiners were trained to identify and annotate background lesions common in the C57BL/6N strain.

As described in prior publications from the IMPC, examinations were carried out in a randomized fashion, and examiners were masked to the genotype and zygosity of mice before and during examination^58^. Each cohort of knockout mice was examined with wild-type animals serving as controls mixed into the cohort. If ocular phenotypes were discovered in a cohort of mice, they were considered to be due to the knockout genotype only if they were exclusively identified in the knockouts. Each examiner was presented with a varying number of mice of different knockout strains on a given examination day, ultimately totaling at least 7 male and 7 female homozygous (viable lines) or heterozygous (non-viable lines) knockout mice and 2 male and 2 female wild-type littermates per knockout strain. At some centers, pupillary light reflexes were evaluated, and the eyelids, third eyelid, conjunctiva, sclera, cornea, iris, and anterior chamber were examined using broad beam illumination at the highest intensity setting (Kowa SL-15, Kowa, Tokyo, Japan) with magnification set at 16×. The irides of all mice were then pharmacologically dilated with a solution of 1:7 10% phenylephrine HCl (Akorn Inc., Lake Forest, IL, USA): 1% tropicamide (Bausch & Lomb Inc., Tampa, FL, USA). The anterior segment was examined using a 0.1 mm slit beam at the highest intensity setting to evaluate the cornea, anterior chamber, and lens, followed by posterior segment evaluation including the vitreous chamber. Fundus examination was performed via indirect ophthalmoscopy using a 60 diopter double aspheric handheld lens (Volk Optical Inc, Mentor, OH, USA).

Background lesions were identified based on expected changes associated with the strain-specific C57BL/6N and retinal degeneration 8 (*rd8*) mutations^59^. Findings in both categories were excluded from the study’s dataset. Background lesions occurred at approximately equal frequencies among knockout and wild-type strains^60^.

### Histology

Mice were euthanized according to IMPC protocols and both eyes were immediately collected and immersion fixed in 10% neutral-buffered formalin. Parasagittal sections were processed routinely, embedded in paraffin, sectioned (4–5 µm), and stained with hematoxylin and eosin. The histopathology was evaluated by a veterinary pathologist. Focal retinal dysplasia observed during histopathology analsysis were considered background strain changes attributed to C57BL/6N genetic background and were not included as genotype-associated findings.

### Ocular imaging

At some centers, mice with an ocular lesion suspected to be related to genotype were flagged for further examination using advanced imaging techniques. These mice were anesthetized with an intraperitoneal injection of ketamine/midazolam (50–75/1–2 mg/kg). Eyes were dilated with tropicamide 1% and phenylephrine 2.5% drops and lubricated with artificial tears containing methylcellulose. Slit lamp anterior segment and fundus images were acquired with a Micron III or IV retinal imaging microscope (Phoenix Research Laboratories).

## Supplementary information

Supplementary Dataset 1
