# Supplementary material for: Genome-wide screening of mouse knockouts reveals novel genes required for normal integumentary and oculocutaneous structure and function
Source: Sci Rep. 2019 Aug 1;9:11211. doi: 10.1038/s41598-019-47286-2 (PMC6672016; doi:10.1038/s41598-019-47286-2)
Supplement: Supplementary file 1 — Supplementary Dataset 1 [file 41598_2019_47286_MOESM1_ESM.docx]

Genome-wide screening of mouse knockouts reveals novel genes required for normal integumentary and oculocutaneous structure and function

Bret A. Moore^1^, Ann M. Flenniken^2,3^, Dave Clary^4^, Ata S. Moshiri^5^, Lauryl M. J. Nutter^2,6^, Zorana Berberovic^2,3^, Celeste Owen^2,3^, Susan Newbigging^2,6^, Hibret Adissu^2,6^, Mohammad Eskandarian^2,3^, Colin McKerlie^2^, International Mouse Phenotyping Consortium, Sara M. Thomasy^7,8^, K.C. Kent Lloyd^4^, Christopher J. Murphy^7,8^, Ala Moshiri^8*^

^1^William R. Pritchard Veterinary Medical Teaching Hospital, School of Veterinary Medicine, U.C. Davis, Davis, CA, United States

^2^The Centre for Phenogenomics, Toronto, ON, M5T 3H7, Canada

^3^Lunenfeld-Tanenbaum Research Institute, Mount Sinai Hospital, Toronto, ON, M5G 1X5, Canada

^4^Mouse Biology Program, U.C. Davis, Davis, CA, United States.

^5^ Division of Dermatology, Department of Medicine, University of Washington, Seattle, WA, United States.

^6^The Hospital for Sick Children, Toronto, ON, M5G 1X8, Canada

^7^Department of Surgical and Radiological Sciences, School of Veterinary Medicine, University of California-Davis, Davis, CA, United States

^8^Department of Ophthalmology & Vision Science, School of Medicine, U.C. Davis, Sacramento, CA, United States.

*Corresponding author:

Email: amoshiri@ucdavis.edu (AM)

**SUPPLEMENTARY MATERIAL**

Supplemental Table 1. Knockout mice with integumentary phenotypes. An example of a published mouse and human integument abnormality associated with each gene is listed if available (with PMID provided), as well as whether one or more (non-KOMP) targeted mutations have been studied for each gene. Specific integument phenotypic findings, other systemic phenotypes associated with the genetic mutation (see below for description of numerical code), and zygosity of the knockout mice from the screening in the IMPC pipeline as also presented. Specific phenotypes described are only for the zygosity displayed (e.g. if heterozygote needed to be bred, homogyous lethality assumed and embryonic phenotypes are not listed). A total of 307 genes were found with an integument phenotype, 81 of which have been previously reported in the literature to affect mouse or human integument. Additionally, for 134 of these 307 genes, one or more knockout mutations have previously been published, but 76 off these were not reported to show aberrant integument phenotypes. In total, 226 knockouts represent potential novel models of integument disease.

1 = Endocrine/exocrine 11 = Respiratory

2 = Taste/olfaction 12 = Cardiovascular

3 = Ear, hearing, vestibular 13 = Behavior, Neurologic

4 = Craniofacial 14 = Metabolism/homeostasis

5 = Muscle phenotye 15 = Growth and Body Size

6 = Immune/Hematopoietic 16 = Reproductive System

7 = Skeletal Phenotye 17 = Embryonic

8 = Limbs, digits, tail 18 = Mortality, aging

9 = Urinary 19 = Eyes

10 = Digestive

| Gene | Gene Name | Mouse Eye PMID | Human Eye PMID | Knock-out Mouse | Integument Phenotype | Other Systemic Phenotypes | Zygosity |
| --- | --- | --- | --- | --- | --- | --- | --- |
| *1700008O03Rik* | RIKEN cDNA 1700008O03 | - | - | - | Abnormal Hair Growth | None | Hom |
| *1700029l15Rik* | RIKEN cDNA 1700029l15 | - | - | - | Abnormal Skin Morphololgy | 16 | Hom |
| *4933402N03Rik* | RIKEN cDNA 4933402N03 | - | - | - | Abnormal Hair Growth | 4, 7, 14 | Hom |
| *Abcb6* | ATP-binding cassette, sub-family B, member 6 | - | Dyschromatosis Universalis 23519333 | 22294697 | Abnormal Vibrissae Morphology | 14 | Hom |
| *Abcd4* | ATP-binding cassette, sub-family B, member 4 | - | Melanoma 17885581 | - | Abnormal Tactile Response | 13 | Hom |
| *Ablim1* | Actin-binding LIM protein 1 | - | - | 12849746 | Decreased Thermal Nociceptive Threshold | 7, 12, 13, 14, 15, 19 | Hom |
| *Acadm* | Acyl-Coenzyme A dehydrogenase, medium chain | - | - | - | Abnormal Vibrissae Morphology | None | Hom |
| *Acer1* | Alkaline ceramidase 1 | Hair Loss 29056331 | - | 29056331 | Abnormal Hair Growth | 6, 7, 14 | Hom |
| *Acsm2* | Acyl-CoA synthetase medium-chain family member 2 | - | - | - | Abnormal Coat Appearance | 13, 14 | Hom |
| *Actg1* | Actin, gamma, cytoplasmic 1 | - | Skin Cancer 28727228 | 16950128 | Pallor | 15, 17 | Hom |
| *Actrt3* | Actin related protein T3 | - | - | - | Abnormal Hair Morphology | 13 | Hom |
| *Acvr1b* | Activin A receptor, type 1B | Hair Loss 21191412 | - | 9512518 | Abnormal Hair Morphology | 1, 7, 13, 15, 16, 17 | Het |
| *Acvr2b* | Activin A receptor, type 2B | - | Systemic Sclerosis 28211533 | 10452853 | Pallor | 13, 15, 17, 19 | Het |
| *Adamtsl4* | ADAMTS-like 4 | - | - | 26405179 | Abnormal Hair Morphology | 13 | Hom |
| *Adar* | Adenosine deaminase, RNA-specific | - | - | - | Pallor | 13, 15, 17, 18 | Hom |
| *Adarb2* | Adenosine deaminase, RNA-specific, B2 | - | - | - | Abnormal Hair Morphology | 1, 6, 9, 13, 16 | Hom |
| *Afap1l2* | Actin filament associated protein 1-like 2 | - | - | 25272040 | Abnormal Hair Morphology | 6, 7, 8, 12, 13, 14 | Hom |
| *Aif1l* | Allograft inflammatory factor 1-like | - | - | - | Abnormal Skin Morphology | 1, 6 | Hom |
| *Aldh2* | Aldehyde dehydrogenase 2, mitochondrial | - | Skin Flushing 10091951 | 10913633 | Abnormal Tactile Response | 6, 7, 13 | Hom |
| *Alg1* | Asparagine-linked glycosylation 1 (beta-1,4-mannosyltransferase) | - | Skin Glycosylation 14973778 | - | Abnormal Hair Pigmentation | None | Het |
| *Alg10b* | ALG10B, alpha-1, 2-Glucosyltransferase | - | - | 24303013 | Skin Bleb | 3, 4, 8, 12, 13, 14, 15, 17, 18, 19 | Hom |
| *Alg8* | Asparagine-linked glycosylation 8 | - | Wrinkled Skin 24555185 | - | Absent Vibrissae | 7, 14 | Het |
| *Anapc15* | Anaphase promoting complex subunit 15 | - | - | - | Abnormal Skin Coloration | 4, 13 ,14, 15, 17, 18, 19 | Hom |
| *Ankle1* | Ankyrin repeat and LEM domain contaning 1 | Psoriasis 28146080 | - | 28146080 | Abnormal Hair Pigmentation | None | Hom |
| *Anks4b* | Ankyrin repeat and sterile alpha motif domain containing 4B | - | - | - | Abnormal Vibrissae Morphology | None | Hom |
| *Ano7* | Anoctamin 7 | - | - | - | Abnormal Hair Morphology | 13 | Hom |
| *Apbb2* | Amyloid beta precursor protein-binding, family B, member 2 | - | - | 16407979 | Abnormal Skin Morphology | 6, 7, 13, 14, 19 | Het |
| *Aph1c* | Aph1 homolog C, gamma secretase subunit | - | - | 15665098 | Abnormal Hair Pigmentation | None | Hom |
| *Apof* | Apolipoprotein F | - | - | 24529150 | Abnormal Hair Morphology | None | Hom |
| *Arf2* | ADP ribosylation factor 2 | - | - | - | Abnormal Hair Pigmentation | 6 | Hom |
| *Arhgef4* | Rho guanine nucleotide exchange factor 4 | - | - | 19897489 | Abnormal Vibrissae Morphology | 6, 7, 13, 14, 15 | Hom |
| *Arih2* | Ariadne RBR E3 ubiquitin protein ligase 2 | - | - | 23179078 | Thick Skin | 1, 7, 9, 12, 13, 14, 15, 16, 17 | Het |
| *Arpc2* | Actin related protein 2/3 complex, subunit 2 | - | Melanoma 25677173 | 25890209 | Abnormal Hair Coloration | 7, 14, 15 | Het |
| *Arpc4* | Actin related protein 2/3 complex, subunit 4 | Psoriasis 29113991 | - | 29113991 | Abnormal Hair Texture | 6, 14 | Het |
| *Art1* | ADP-ribosyltransferase 1 | - | - | 9300695 | Abnormal Skin Morphology | 6 | Hom |
| *Ash1l* | ASH1 like histone lysine methyltransferase | Epidermal Hyperplasia 28374742 | - | 24012418 | Abnormal Hair Appearance | 12, 13, 19 | Het |
| *Aspg* | Asparaginase | - | - | - | Abnormal Skin Morphology | 14 | Hom |
| *Atf6b* | Activating transcription factor 6 beta | - | - | 17765680 | Scaly Skin | None | Het |
| *Atn1* | Atrophin 1 | - | - | 17150957 | Abnormal Skin Morphology | 6, 12, 13, 14 | Hom |
| *Atp2b1* | ATPase, Ca++ transporting, plasma membrane 1 | - | - | 151178683 | Abnormal Vibrissae Morphology | 1, 14, 15, 16, 17 | Het |
| *B2m* | Beta-2 microglobulin | Bullous Pemphigoid 9271593 | Cutaneous Amyloidosis 11874487 | 8013958 | Abnormal Skin Morphology | 13, 15, 16, 17 | Het |
| *B9d1* | B9 protein domain 1 | - | - | 22179047 | Abnormal Tactile Response | 13, 14, 15 | Het |
| *Barx2* | BarH-like homeobox 2 | Hair Follicle Development 15728386 | - | 21750040 | Abnormal Hair Pigmentation, Absent Vibrissae | 8, 12, 13, 18, 19 | Hom |
| *Baz2a* | Bromodomain adjacent to zinc finger domain, 2A | - | - | - | Abnormal Skin Morphology | 1, 6, 9, 14, 16 | Hom |
| *Bend3* | BEN domain containing 3 | - | - | 25457167 | Abnormal Hair Pigmentation | 13 | Het |
| *Bex2* | Brain expressed X-linked 2 | - | - | 25143383 | Abnormal Hair Pigmentation | 6, 13, 15, 19 | Hom |
| *Bms1* | BMS1, ribosome biogenesis factor | - | Aplasia Cutis Congenita 23785305 | - | Abnormal Hair Pigmentation | 19 | Het |
| *Brd7* | Bromodomain containing 7 | - | - | 25721744 | Abnormal Hair Morphology | 13, 14, 15 | Het |
| *Brwd1* | Bromodomain and WD repeat domain containing 1 | - | - | 30250168 | Abnormal Skin Morphology | 1, 6, 16 | Hom |
| *C530008M17Rik* | RIKEN cDNA C530008M17 | - | - | - | Abnormal Hair Morphology | 7 | Hom |
| *Cabp1* | Calcium binding protein 1 | - | - | 24631676 | Abnormal Hair Appearance | 14 | Hom |
| *Cadm1* | Cell adhesion molecule 1 | Impaired Wound Healing 19783739 | Autoimmune Alopecia, Skin Cancer  22210910 | 197833739 | Abnormal Hair Morphology | 6, 13, 15 | Hom |
| *Carmil2* | Capping protein regulator and myosin 1 linker 2 | - | Dermatitis 29479355 | - | Abnormal Hair Pigmentation | 19 | Hom |
| *Caskin1* | CASK interacting protein 1 | - | - | 30359304 | Abnormal Hair Pigmentation | 1, 9, 14, 16 | Het |
| *Cast* | Calpastatin | - | Leukonychia 25683118 | 15691848 | Abnormal Nail Morphology | 7, 8, 13, 14 | Hom |
| *Castor1* | Cytosolic arginine sensor for mTORC1 subunit 1 | - | - | - | Abnormal Skin Morphology | 6, 13, 14 | Hom |
| *Ccdc77* | Coiled-coil domain containing 77 | - | - | - | Abnormal Hair Growth | 14 | Hom |
| *Cd109* | CD109 antigen | Epidermal Hyperplasia 22846721 | - | 22846721 | Absent Vibrissae | None | Hom |
| *Cd248* | CD248 antigen, endosialin | - | Skin Cancer 16420310 | 16492758 | Abnormal Skin Morphology | 1, 6, 14, 15, 16 | Hom |
| *Cd5* | CD5 antigen | - | - | - | Abnormal Skin Morphology | 6, 13 | Hom |
| *Cd5l* | CD5 antigen-like | - | Atopic Dermatitis 19116453 | 9892623 | Abnormal Nail Morphology | 13 | Hom |
| *Cdc123* | Cell division cycle 123 | - | - | - | Abnormal Hair Pigmentation | 13 | Het |
| *Cdc34* | Cell division cycle 34 | - | - | - | Abnormal Nail Morphology | 6 | Het |
| *Cdc42ep5* | CDC42 effector protein 5 | - | - | - | Abnormal Hair Pigmentation | None | Hom |
| *Cdc7* | Cell division cycle 7 | - | Melanoma 19278428 | 11980714 | Abnormal Skin Morphology | 6 | Het |
| *Cdk9* | Cyclin-dependent kinase 9 | - | - | 30209133 | Abnormal Skin Morphology | 12, 13, 14, 16 | Het |
| *Cep97* | Centrosomal protein 97 | - | - | - | Bleb | None | Hom |
| *Ces1f* | Carboxylesterase 1F | - | - | - | Abnormal Skin Coloration | 3 | Hom |
| *Chchd7* | Coiled-coil-helix-coiled-coil-helix domain containing 7 | - | - | - | Abnormal Skin Morphology | 6, 11 | Hom |
| *Chic2* | Cysteine-rich hydrophobic domain 2 | - | - | - | Abnormal Hair Pigmentation | 19 | Het |
| *Chst8* | Carbohydrate sulfotransferase 8 | - | Peeling Skin Syndrome 22289416 | - | Abnormal Vibrissae Morphology | 1, 13, 16, 19 | Hom |
| *Chsy3* | Chondroitin sulfate synthase 3 | - | - | - | Thick Skin | 1, 7, 9, 12, 13, 15, 16 | Hom |
| *Ckb* | Creatine kinase, brain | - | - | 18724377 | Abnormal Vibrissae Morphology | 3, 6, 7, 13, 14, 15 | Hom |
| *Clk1* | CDC-like kinase 1 | - | - | - | Abnormal Tactile Response | 6, 7, 13, 14, 15, 19 | Hom |
| *Clrn1* | Clarin 1 | - | - | 19680541 | Abnormal Vibrissae Morphology | 3, 13 | Hom |
| *Cnga3* | Cyclic nucleotide gated channel alpha 3 | - | - | 10377453 | Abnormal Skin Morphology | 6, 12, 13, 16 | Hom/Het |
| *Cog6* | Component of oligomeric golgi complex 6 | - | Psoriasis 20605848 | - | Abnormal Hair Pigmentation | 6, 7, 14, 19 | Hom |
| *Cotl1* | Coactosin-like 1 (Dictyostelium) | - | - | - | Abnormal Hair Pigmentation | 19 | Hom |
| *Cox6b1* | Cytochrome c oxidase, subunit 6B1 | - | - | - | Abnormal Skin Morphology | 1, 7, 8, 18, 19 | Het/Hom |
| *Cpvl* | Carboxypeptidase, vitellogenic-like | - | - | - | Thick Skin | 6, 14 | Hom |
| *Crb2* | Crumbs family member 2 | - | - | 23001562 | Abnormal Vibrissae Morphology | None | Het |
| *Cstdc2* | RIKEN cDNA 9230104L09 | - | - | - | Abnormal Skin Morphology | 13, 14 | Hom |
| *Ctso* | Cathepsin o | - | - | - | Abnormal Vibrissae Morphology | 14 | Hom |
| *Cxcl17* | C-X-C motif chemokine ligand 17 | - | Psoriasis 28389593 | 14973458 | Abnormal Skin Morphology, Abnormal Hair Pigmentation | 12, 13 | Hom |
| *Cyp21a1* | Cytochrome P450, family 21, subfamily a, polypeptide 1 | - | - | - | Abnormal Skin Morphology | 6, 13 | Het |
| *Cyp2d22* | Cytochrome P450, family 2, subfamily d, polypeptide 22 | - | - | - | Abnormal Skin Morphology | 6 | Hom |
| *D430041D05Rik* | RIKEN cDNA D430041D05 gene | - | - | - | Abnormal Hair Pigmentation | 7, 13, 14, 15 | Hom |
| *Dbn1* | Debrin 1 | - | Skin Tumors 16185277 | 19711416 | Pallor | 6, 7, 13, 14, 15, 19 | Het |
| *Dcp2* | Decapping MRNA 2 | - | - | 21070968 | Abnormal Hair Pigmentation | 12, 19 | Het |
| *Dct* | Dopachrome tautomerase | Pigmentation 15060160 | Hermansky Pudlak Syndrome 15632015 | 15060160 | Abnormal Skin Morphology, Abnormal Hair Pigmentation | 14 | Hom |
| *Ddx39* | DEAD box polypeptide 39 | - | - | - | Abnormal Hair Appearance | 14 | Het |
| *Ddx55* | DEAD box polypeptide 55 | - | - | - | Abnormal Hair Appearance | 14 | Het |
| *Ddx59* | DEAD-box helicase 59 | - | - | - | Abnormal Hair Pigmentation | 6 | Het |
| *Def6* | Differently expressed in FDCP 6 | - | Psoriasis 22528235 | 19915062 | Abnormal Hair Growth | 6, 7 | Hom |
| *Dgat2l6* | Diacylglycerol O-acyltransferase 2-like 6 | - | Sebum production 30895322 | - | Absent Vibrissae | 12 | Hom |
| *Dnah17* | Dynein, axonemal, heavy chain 17 | - | - | - | Abnormal Hair Growth | 16 | Hom |
| *Dnajb7* | DnaJ heat shock protein family member B7 | - | - | - | Abnormal Skin Morphology | 14 | Hom |
| *Dnase1l2* | Deoxyribonuclease 1-like 2 | Parakeratosis 28743926 | - | 28743926 | Abnormal Nail Morphology | 4, 7, 8, 12, 13, 14, 15, 16, 19 | Hom |
| *Dnm1l* | Dynamin 1-like | - | Skin Cancer 26032958 | 19752021 | Abnormal Hair Morphology | 19 | Het |
| *Dop1b* | DOP1 leucine zipper like protein B | - | - | - | Abnormal Hair Appearance | 7, 13, 14 | Hom |
| *Dph6* | Diphthamine biosynthesis 6 | - | - | - | Abnormal Hair Pigmentation | 6, 7, 8, 15 | Hom |
| *Dpm3* | Dolichyl-phosphate mannosyltransferase polypeptide 3 | - | - | - | Bleb | 1, 13, 16 | Het |
| *Dram2* | DNA damage regulated autophagy modulator 2 | - | - | - | Abnormal Hair Pigmentation | 13 | Hom |
| *Dsg1b* | Desmoglein 1 beta | - | Keratoderma 29229434 | - | Abnormal Hair Appearance | 7, 14, 15, 19 | Hom |
| *Dtnbp1* | Dystrobrevin binding protein 1 | Decreased Pigmentation 12923531 | Hermansky Pudlak Syndrome 12923531 | 12923531 | Abnormal Hair Pigmentation | 13, 14, 19 | Hom |
| *Duoxa2* | Dual oxidase maturation factor 2 | - | - | 22301785 | Abnormal Hair Growth | 4, 6, 7, 8, 12, 13, 15, 18, 19 | Hom |
| *Dusp7* | Dual specificity phosphatase 7 | - | - | - | Abnormal Hair Pigmentation | 14, 16 | Hom |
| *Edem3* | ER degradation enhancer, mannosidase alpha-like 3 | - | - | - | Abnormal Hair Pigmentation | 7 | Het |
| *Ehbp1l1* | EH domain binding protein 1-like 1 | - | - | 26833786 | Abnormal Hair Growth | None | Het |
| *Eif2s2* | Eukaryotic translation initiation factor 2, subunit 2 | - | Skin Pigmentation 25963972 | - | Abnormal Hair Appearance | 6, 12 | Het |
| *Eif4enif1* | Eukaryotic translation initiation factor 4E nuclear import factor 1 | - | - | - | Abnormal Skin Coloration | 1,9, 12, 14, 15, 17 | Hom/Het |
| *Elmod1* | ELMO/CED-12 domain containing 1 | - | - | 29222402 | Abnormal Tactile Response | 3, 6, 7, 12, 13, 14, 15, 19 | Hom |
| *Emc4* | ER membrane protein complex subunit 4 | - | - | - | Abnormal Hair Pigmentation | None | Het |
| *Endog* | Endonuclease G | - | - | 14663139 | Abnormal Hair Pigmentation, Abnormal Skin Morphology | 1, 16 | Hom |
| *Epha6* | Eph receptor A6 | - | - | 18450376 | Abnormal Skin Morphology | 1, 6, 9, 13, 16 | Hom |
| *Erlin2* | ER lipid raft associated 2 | - | - | - | Abnormal Hair Growth | 6, 7 | Hom |
| *Exoc6* | Exocyst complex component 6 | - | - | - | Abnormal Skin Appearance | 7, 8, 15, 17, 18 | Het/Hom |
| *Fam107b* | Family with sequence similarity 107, member b | - | - | - | Abnormal Hair Morphology | 6, 15 | Hom |
| *Fancl* | Fanconi anemia complementation group L | - | - | - | Abnormal Hair Pigmentation | 7, 14 | Hom |
| *Far2* | Fatty acyl CoA reductase 2 | Skin, Hair Abnormalities 30372477 | - | 30372477 | Abnormal Hair Appearance | 12, 13, 14, 15 | Hom |
| *Fcrla* | Fc receptor-like A | - | - | 20668221 | Abnormal Hair Pigmentation | 6, 13 | Hom |
| *Fgf11* | Fibroblast growth factor 11 | - | - | - | Abnormal Tactile Response | 6, 13 | Hom |
| *Ficd* | FIC domain containing | - | - | - | Absent Vibrissae | None | Hom |
| *Fmnl3* | Formin like 3 | - | - | - | Abnormal Skin Coloration | None | Het |
| *Fndc3b* | Fibronectin type III domain containing 3B | - | - | - | Abnormal Hair Pigmentation | 9, 12, 15, 16, 17, 19 | Het |
| *Fosb* | FBJ osteosarcoma oncogene B | - | - | 8668344 | Abnormal Skin Morphology | 9, 12 | Hom |
| *Foxn1* | Forkhead box N1 | Wound Healing 29637306 | Alopecia 24432845 | 29637306 | Abnormal Skin Morphology | 1, 6, 13, 16 | Hom/Het |
| *Fryl* | FRY like transcription coactivator | - | - | 29409347 | Abnormal Skin Pigmentation | 14 | Het |
| *Furin* | Paired basic animo acid cleaving enzyme | - | Skin Cancer 23441131 | 9811571 | Abnormal Vibrissae Morphology | 8 | Het |
| *Gcdh* | Glutaryl-CoA dehydrogenase | - | - | 26607633 | Abnormal Coat Appearance, Abnormal Skin Coloration | 13 | Het |
| *Gdap1* | Ganglioside-induced differentiation-associated-protein 1 | - | Charcot Marie Tooth Disease 26362287 | 24480485 | Abnormal Skin Morphology | 1, 6, 12, 13, 16 | Hom |
| *Ggps1* | Geranylgeranyl diphosphate synthase 1 | - | - | - | Abnormal Hair Morphology | 14, 19 | Het |
| *Gp6* | Glycoprotein 6 | - | - | 17991808 | Abnormal Vibrissae Morphology | 6, 19 | Hom |
| *Gpr34* | G protein-coupled receptor 34 | - | - | 21097509 | Absent Vibrissae | 1, 16 | Hom |
| *Gpr65* | G protein-coupled receptor 65 | - | Skin Cancer 30339292 | 27287411 | Abnormal Skin Morphology | 12, 13, 16 | Hom |
| *Gsta4* | Glutathione S-transferase alpha 4 | Skin Cancers 20966433 | Skin Cancers 20966433 | 27998724 | Abnormal Hair Pigmentation | 12 | Hom |
| *Gys2* | Glycogen synthase 2 | - | - | 20178984 | Abnormal Vibrissae Morphology | 6, 14, 15 | Hom |
| *Hdgfl3* | HDGF like 3 | - | - | - | Abnormal Skin Morphology | 13, 19 | Hom |
| *Hectd1* | HECT domain E3 ubiquitin protein ligase 1 | - | - | - | Abnormal Hair Pigmentation | None | Het |
| *Hyal3* | Hyaluronoglucosaminidase 3 | - | - | 18762256 | Abnormal Hair Appearance | 14 | Hom |
| *Icam5* | Intercellular adhesion molecule 5, telencephalin | - | - | 11135016 | Abnormal Vibrissae Morphology | None | Hom |
| *Igfbp3* | Insulin like growth factor binding protein 3 | - | Skin Cancer 15727632 | 28088287 | Abnormal Hair Pigmentation | 7, 8, 14 | Hom |
| *Il18rap* | Intercellular adhesion molecule 5, telencephalin | - | - | 15843532 | Abnormal Vibrissae Morphology | None | Hom |
| *Il33* | Interleukin 33 | - | Atopic Dermatitis 30688372 | 29379874 | Abnormal Hair Morphology | None | Hom |
| *Ing4* | Inhibitor of growth family, member 4 | - | Melanoma 18375955 | - | Abnormal Skin Morphology | 6, 9, 13, 14 | Hom |
| *Insl5* | Insulin-like 5 | - | - | 22822165 | Abnormal Vibrissae Morphology | 11, 13, 14 | Hom |
| *Itsn2* | Intersectin 2 | - | - | 29773874 | Abnormal Hair Morphology | 13 | Hom |
| *Kansl1* | KAT8 regulatory NSL complex subunit 1 | - | - | 28704368 | Abnormal Hair Pigmentation | 13, 14, 19 | Het |
| *Kcnj16* | Potassium inwardly-rectifying channel, subfamily J, member 16 | - | - | 21633011 | Abnormal Tactile Response | 7, 13, 15 | Hom |
| *Kctd17* | Potassium channel tetramerization domain | - | - | - | Absent Vibrissae | None | Hom |
| *Kdm4c* | Lysine specific demethylase 4C | Skin Cancer 26248577 | - | 26248577 | Decreased Thermal Nociceptive Threshold | 6, 13, 14 ,15 | Hom |
| *Kdm8* | Lysine specific demethylase 8 | - | - | 22241836 | Abnormal Tactile Response | 6, 13, 14, 15, 19 | Het |
| *Kif2c* | Kinesin family member 2C | - | - | - | Abnormal Skin Coloration | None | Het |
| *Klhdc2* | Kelch domain containing 2 | - | - | - | Abnormal Skin Morphology | 1, 4, 6, 12, 13, 15, 19 | Het |
| *Krt31* | Keratin 31 | - | Hair Keratin 22215855 | - | Abnormal Hair Appearance | None | Hom |
| *Krtap17-1* | Keratin associated protein 17-1 | - | Atopic Dermatitis 30728947 | - | Abnormal Hair Morphology | 7, 13, 14, 15 | Hom |
| *L3mbtl3* | L3MBTL3 histone methyl-lysine binding protein | - | - | 15889154 | Abnormal Skin Morphology | 13, 14 | Het |
| *Lama4* | Laminin, alpha 4 | - | Skin Development 15086543 | 20035058 | Abnormal Hair Pigmentation, Abnormal Skin Morphology | 12, 14 | Hom |
| *Lor* | Loricrin | Skin Development 11038185 | Keratoderma 27520397 | 11038185 | Abnormal Skin Morphology, Abnormal Vibrissae Morphology | 1, 6, 13, 16, 18 | Hom/Het |
| *Lrig1* | Leucine rich-repeats and immunoglobulin- like domains 1 | Skin Wound Healing 26753643 | - | 26753643 | Abnormal Skin Coloration | 8, 12, 13, 15 | Hom |
| *Lrmp* | Lymphoid restricted membrane protein | - | - | - | Abnormal Hair Appearance | None | Hom |
| *Lst1* | Leukocyte specific transcript 1 | Skin Fibrosis 18580965 | - | 18580965 | Abnormal Skin Morphology | 1, 12, 13, 16 | Hom |
| *Lypd6* | Ly6/PLAUR domain containing 6 | - | - | 29195920 | Thick Skin, Scaly Thin | 1, 12, 13, 16 | Het |
| *Lyst* | Lysosomal trafficking regulator | Hair Pigmentation 16518687 | Chediak Higashi Syndrome 21488161 | 17982137 | Abnormal Hair Pigmentation | 6, 14, 15, 18, 19 | Hom |
| *Maea* | Macrophage erythroblast attacher | - | - | - | Abnormal Hair Appearance | 13 | Het |
| *Mc1r* | Melanocortin 1 receptor | Angelman Syndrome 21733131 | Skin Cancer 29968341 | - | Abnormal Hair Pigmentation, Abnormal Skin Morphology | 6, 12, 13 | Hom |
| *Mcub* | Mitochondrial calcium uniporter dominant negative beta subunit | Skin, Hair Abnormalities 12573256 | - | - | Absent Vibrissae | 13 | Hom |
| *Mdp1* | Magnesium dependent phosphatase 1 | - | - | - | Abnormal Hair Appearance | None | Hom |
| *Meak7* | TBM/LysM associated domain containing 1 | - | - | - | Abnormal Skin Morphology | 11, 16 | Hom |
| *Mettl16* | Methyltransferase like 16 | - | - | - | Abnormal Hair Pigmentation | 15, 17 | Het |
| *Mettl7b* | Methyltransferase like 7B | - | - | - | Abnormal Hair Pigmentation | 13, 14 | Hom |
| *Mfsd8* | Major facilitator superfamily domain containing 8 | - | - | 26681805 | Abnormal Hair Pigmentation | 12, 13, 19 | Hom |
| *Micalcl* | MICAL c-terminal like | - | - | - | Abnormal Skin Morphology | 6, 16 | Hom |
| *Mid2* | Midline 2 | - | - | - | Scaly Skin | 6 | Hem |
| *Mir100* | MicroRNA 100 | - | - | - | Abnormal Hair Pigmentation, Abnormal Skin Coloration | None | Hom |
| *Mmp11* | Matrix metallopeptidase 11 | - | - | 27126782 | Abnormal Skin Coloration | 6, 8, 12, 13, 14 | Hom/Het |
| *Mogs* | Mannosyl-oligosaccharide glucosidase | - | - | - | Abnormal Skin Coloration | 1, 6, 10, 11, 19 | Het |
| *Mplkip* | M-phase specific PLK1 interacting protein | - | Trichothio-dystrophy 29421601 | - | Abnormal Skin Morphology | 1, 9, 12, 16 | Hom |
| *Mpv17l2* | MPV17 mitochondrial inner membrae protein like 2 | - | - | - | Abnormal Hair Pigmentation | 6, 12, 13, 14 | Hom |
| *Mpz* | Myelin protein zero | - | Charcot Marie Tooth Disease 26234237 | 10586252 | Abnormal Skin Morphology | 7, 11, 13, 14, 15 | Hom |
| *Mroh4* | Maestro heat-like repeat family member 4 | - | - | - | Abnormal Hair Appearance and Growth | None | Hom |
| *Mthfd2l* | Methylenetetrahydrofolate dehydrogenase 2-like | - | - | - | Abnormal Skin Morphology | 12 | Hom |
| *Mturn* | Maturin, neural progenitor differentiation regulator homolog | - | - | - | Abnormal Hair Morphology | 7, 15 | Hom |
| *Muc3a* | Mucin 3A, cell surface associated | - | - | - | Abnormal Hair Morphology, Absent Vibrissae | None | Hom |
| *Myh10* | Myosin heavy chain 10 | - | - | 29084269 | Abnormal Skin Coloration | 12, 14, 15, 17, 18 | Hom/Het |
| *Myo10* | Myosin X | Pigmentation 29509981 | Skin Wound Healing 18818677 | 30679680 | Abnormal Hair Pigmentation | 3, 7, 8, 13, 14, 19 | Hom |
| *Myo5a* | Myosin VA | Hair Pigmentation 24721909 | - | 24721909 | Abnormal Hair Pigmentation | 13 | Hom |
| *Mysm1* | Myb like, SWIRM And MPN domains 1 | Hair Pigmentation 24721909 | Panniculitis 30746751 | 27277682 | Abnormal Hair Morphology, Abnormal Hair Pigmentation | 4, 6, 7, 8, 13, 14, 15, 19 | Hom |
| *Nacc1* | Nucleus accumbens associated 1, BEN and BTB domain containing | - | Melanoma 21562571 | - | Absent Vibrissae | 3, 6, 7, 9, 13, 14, 15, 16 | Hom |
| *Nacc2* | Nucleus accumbens associated 2, BEN and BTB domain containing | - | - | - | Abnormal Hair Appearance | 13 | Hom |
| *Nadk2* | NAD kinase 2, mitochondrial | - | - | - | Abnormal Hair Pigmentation | 13, 14, 19 | Het |
| *Nampt* | Nicotinamide phosphoribosyltransferase | - | Melanoma 23051650 | 27229177 | Abnormal Skin Morphology | 12, 13, 15, 17 | Het |
| *Nck1* | Non-catalytic region of tyrosine kinase adaptor protein 1 | - | - | 23280595 | Abnormal Skin Morphology | 12, 19 | Hom |
| *Nctc1* | Non-coding transcript 1 | - | - | - | Abnormal Hair Appearance | None | Hom |
| *Neurl2* | Neuralized E3 ubiquitin protein ligase 2 | - | - | 14960280 | Abnormal Hair Appearance | None | Hom |
| *Nfkb1* | Nuclear factor of kappa light polypeptide gene enhancer in B cells 1 | Skin Fibrosis 23562440 | Psoriasis 29748001 | 28726772 | Abnormal Skin Condition | 6, 14, 15, 19 | Hom |
| *Nudt19* | Nudix-type motif 19 | - | - | 29378847 | Abnormal Skin Morphology | 12 | Hom |
| *Obp2a* | Odorant binding protein 2A | - | - | - | Abnormal Hair Pigmentation | None | Hom |
| *Odf3l2* | Outer dense fiber of sperm tails 3-like 2 | - | - | - | Absent Vibrissae | 3, 13 | Hom |
| *Ogfod1* | 2-oxoglutarate and iron-dependent oxygenase domain containing 1 | - | - | - | Abnormal Skin Condition | 6, 7, 8 | Hom |
| *Ovol1* | Ovo like zinc finger 1 | Skin Development 17049212 | Skin Development 29454536 | 17049212 | Abnormal Vibrissae Morphology | 13 | Het |
| *P3h1* | Prolyl 3-hydroxylase 1 | Skin Collagen Abnormalities 20363744 | - | 20363744 | Thin Skin | 7, 8, 13 ,15 | Hom |
| *Pde6a* | Phosphodiesterase 6A, cGMP-specific, rod, alpha | - | - | - | Abnormal Hair Morphology | 12 | Hom |
| *Pdpn* | Podoplanin | - | - | - | Absent Vibrissae | 14 | Het |
| *Pdzd8* | PDZ domain containing 8 | - | - | - | Abnormal Vibrissae Morphology | 6, 13, 14 | Hom |
| *Pebp1* | Phosphatidylethanolamine bindings protein 1 | - | - | 22926403 | Abnormal Skin Morphology | 16 | Hom |
| *Per2* | Period circadian regulator 2 | Skin Molecular Clock 19037239 | - | 28607147 | Abnormal Hair Pigmentation | 7, 14 | Hom |
| *Phtf1* | Putative homeodomain transcription factor 1 | - | - | - | Abnormal Skin Morphology | 1, 12, 13, 16 | Hom |
| *Plac8* | Placenta-specific 8 | - | - | 30671116 | Abnormal Hair Morphology | 13, 19 | Hom |
| *Plxnb3* | Plexin B3 | - | - | - | Abnormal Hair Morphology | None | Hem |
| *Polr3f* | Polymerase III polypeptide F | - | - | - | Abnormal Hair Growth | 6 | Het |
| *Ppfibp2* | PTPRF interacting protein, binding protein 2 | - | - | - | Abnormal Skin Morphology | 6 | Hom |
| *Ppl* | Periplakin | Epidermal Abnormalities 18166659 | Epidermal Abnormalities 24352042 | 18166659 | Abnormal Skin Morphology | 6, 9 | Hom |
| *Ppp1r32* | Protein phosphatase 1 regulatory subunit 32 | - | - | - | Abnormal Hair Pigmentation | 13 | Hom |
| *Ppp5c* | Protein phosphatase 5 catalytic subunit | - | - | 24220247 | Abnormal Hair Pigmentation | 6, 7, 14, 15 | Hom |
| *Prdm5* | PR domain containing 5 | - | - | 23873026 | Abnormal Skin Morphology | 1, 3, 12, 13, 14, 16, 19 | Hom |
| *Prodh* | Proline dehydrogenase | - | - | - | Abnormal Hair Pigmentation | 7, 8, 13 | Hom |
| *Prom2* | Prominin 2 | - | - | - | Abnormal Nail Morphology | 6, 13, 14 | Hom |
| *Prss53* | Protease, serine 53 | - | Hair Color and Thickness 26926045 | - | Abnormal Hair Growth, Abnormal Vibrissae Morphlogy | 6, 13 | Hom |
| *Ptprd* | Protein tyrosine phosphatase, receptor type D | - | Skin Cancer 17420988 | 26181631 | Abnormal Hair Appearance | 6, 7, 13, 14, 15, 18 | Hom |
| *Rab24* | RAB24 member RAS oncogene family | - | - | - | Abnormal Hair Appearance | 7, 13, 15 | Hom |
| *Rab39b* | RAB39B member RAS oncogene family | - | - | - | Abnormal Skin Morphology | 13 | Hem |
| *Rabl2* | RAB, member RAS oncogene family-like 2 | - | - | 27732084 | Abnormal Nail Morphology | 8, 16 | Hom |
| *Rad18* | RAD18 E3 ubiquitin protein ligase | - | Skin Cancer 29859927 | 28082021 | Abnormal Hair Morphology and Texture | 14, 15 | Hom |
| *Rasal2* | RAS protein activator like 2 | - | - | - | Abnormal Hair Appearance and Growth | 13 | Hom |
| *Rassf8* | Ras association domain family member 8 | - | Melanoma 26334503 | - | Abnormal Hair Morphology | 1, 12, 16 | Het |
| *Rbms1* | RNA binding motif, single stranded interacting protein 1 | - | - | - | Abnormal Hair Morphology | 12, 15 | Het |
| *Rcc2* | Regulator of chromosome condensation 2 | - | - | - | Abnormal Hair Pigmentation | 6, 12, 14, 19 | Hom |
| *Recql4* | RecQ protein-like 4 | Skin Abnormalities 12915449 | Rothmund-Thomson Syndrome 27247962 | 12915449 | Abnormal Hair Morphology | 13, 14, 15 | Het |
| *Rel* | Reticuloendotheliosis oncogene | - | - | - | Absent Vibrissae | 6, 13 | Hom |
| *Rhbdl1* | Rhomboid like 1 | - | - | - | Abnormal Skin Morphology | 14, 15, 17 | Het |
| *Rhd* | Rh blood group, D antigen | - | - | 19807729 | Abnormal Skin Condition | 6, 14 | Hom |
| *Riiad1* | Regulatory subunit of type II PKA R-subunit domain containing 1 | - | - | - | Absent Vibrissae | 7, 13 | Het |
| *Ripk3* | Receptor-interacting serine-threonine kinase 3 | Skin Wound Healing 26451737 | - | 30842945 | Absent Vibrissae | None | Hom |
| *Rln3* | Relaxin 3 | - | - | 26023064 | Abnormal Hair Pigmentation | 7 | Hom |
| *Rnase1* | Ribonuclease, RNase A family, 1 | - | - | - | Abnormal Skin Morphology | 6, 13 | Hom |
| *Rnaseh2b* | Ribonuclease H2, subunit B | - | - | - | Abnormal Vibrissae Morphology | None | Het |
| *Rnf135* | Ring finger protein 135 | - | - | - | Abnormal Skin Morphology | 10 | Hom |
| *Rnf7* | Ring finger protein 7 | - | - | - | Abnormal Tactile Response | 13, 14 | Het |
| *Rspo1* | R-spondin 1 | - | Keratoderma 29575617 | 27611670 | Abnormal Vibrissae Morphology | 1, 3, 6, 9, 13, 14, 15, 16, 19 | Hom |
| *Rttn* | Rotatin | - | - | - | Abnormal Skin Morphology | 6, 15, 17 | Het |
| *Sacs* | Sacsin | - | - | 25260547 | Abnormal Skin Morphology | 6, 7, 9, 14, 16, 18 | Hom/Het |
| *Sar1b* | Secretin associated Ras related GTPase 1B | - | - | - | Abnormal Skin Morphology | 14 | Het |
| *Sbno1* | Strawberry notch homolog 1 | - | - | - | Abnormal Coat Appearance, Abnormal Hair Pigmentation | 6, 7, 8, 12 | Het |
| *Sdc2* | Syndecan 2 | - | - | - | Abnormal Hair Pigmentation | 6, 14, 19 | Hom |
| *Sdr42e1* | Short chain dehydrogenase/reductase family 42E, member 1 | - | - | - | Abnormal Hair Morphology | 19 | Hom |
| *Sec63* | SEC63-like | - | - | 25844898 | Abnormal Vibrissae Morphology, Absent Vibrissae | 6, 14 | Het |
| *Sema3f* | Sema domain, immunoglobulin domain, short basic domain, secreted 3F | Dermal Lymphatic Network 26319580 | - | 26856818 | Abnormal Nail Morphology | 7, 13, 14, 15, 18, 19 | Hom |
| *Senp7* | SUMO1/sentrin specific peptidase 7 | - | - | - | Abnormal Hair Pigmentation | None | Hom |
| *Setd5* | SET domain containing 5 | - | - | 27864380 | Abnormal Hair Pigmentation | 4, 6, 7, 13, 14, 15 | Het |
| *Sgip1* | SH3-domain GRB2-like interacting protein 1 | - | - | - | Abnormal Nail Morphology | 8, 12, 13, 19 | Hom/Het |
| *Shprh* | SNF2 histone linker PHD RING helicase | - | - | - | Abnormal Skin Morphology | 6, 14 | Hom |
| *Sik3* | SIK family kinase 3 | - | - | 25619259 | Abnormal Hair Pigmentation | 4, 6, 7, 8, 14, 15, 16, 19 | Hom |
| *Slc17a9* | Solute carrier family 17 member 9 | - | Actinic Porokeratosis [25180256](https://www.ncbi.nlm.nih.gov/pubmed/25180256) | - | Abnormal Skin Morphology | 13, 19 | Hom |
| *Slc24a5* | Solute carrier family 24 member 5 | - | Skin Coloration 28300201 | 18424845 | Abnormal Hair Pigmentation | 13, 14, 19 | Hom |
| *Slc35c2* | Solute carrier family 35 member C2 | - | - | - | Abnormal Hair Morphology | None | Hom |
| *Slc38a10* | Solute carrier family 38 member 10 | - | - | - | Abnormal Tactile Response | 5, 6, 7, 8, 12, 13, 14, 15, 19 | Hom |
| *Slc46a1* | Solute carrier family 46 member 1 | - | - | - | Abnormal Hair Appearance | 6, 14 | Het |
| *Slc46a3* | Solute carrier family 46 member 3 | - | - | - | Abnormal Hair Appearance | 6, 13 | Hom |
| *Slc52a2* | Solute carrier family 52 member 2 | - | - | - | Absent Vibrissae | 1, 6, 7, 8 | Het |
| *Slc52a3* | Solute carrier family 52 member 3 | - | - | 27272163 | Abnormal Vibrissae Morphology | 14 | Het |
| *Slc5a7* | Solute carrier family 5 member 7 | - | - | - | Abnormal Hair Growth | 6, 13 | Het |
| *Slc8b1* | Solute carrier family 8 member B1 | - | - | - | Abnormal Hair Appearance | 13, 14 | Hom |
| *Slu7* | SLU7 splicing factor homolog | - | - | - | Abnormal Hair Growth | 6 | Het |
| *Smc3* | Structural maintenance of chromosomes 3 | - | Cornelia de Lange Syndrome 21165303 | - | Abnormal Hair Coloration | 4, 6, 7, 14, 15 | Het |
| *Smoc1* | SPARC related modular calcium binding 1 | - | - | 21750680 | Abnormal Hair Pigmentation | 6, 13, 15 | Het |
| *Smpdl3a* | Sphingomyelin phosphodiesterase, acid-like 3A | - | - | - | Abnormal Skin Morphology | 14 | Hom |
| *Sparc* | Secreted protein acidic and cysteine rich | Dermal Collagen Abnormalities 12787119 | - | 30626728 | Abnormal Hair Pigmentation | 4, 7, 8, 12, 15, 19 | Hom |
| *Sptssb* | Serine palmitoyltransferase, small subunit B | - | - | - | Abnormal Hair Appearance | 9, 14 | Hom |
| *Stx8* | Syntaxin 8 | - | - | - | Alopecia, Abnormal Tactile Response | 7, 12, 13, 14, 15 | Hom |
| *Svep1* | Sushi, von Willebrand factor type A, EGF and pentraxin domain containing 1 | Epidermal Development 27892606 | - | 27892606 | Abnormal Skin Appearance | 6, 8, 14, 18 | Hom/Het |
| *Tanc1* | Tetratricopeptide repeat, ankyrin repeat and coiled-coil containing 1 | - | - | - | Abnormal Skin Morphology | 9 | Hom |
| *Tbc1d30* | TBC domain family, member 30 | - | - | - | Abnormal Hair Morphology | 7, 13 | Hom |
| *Tceal3* | Transcription elongation factor A-like 3 | - | - | - | Abnormal Skin Morphology | 6, 9 | Hem |
| *Tceal9* | Transcription elongation factor A-like 9 | - | - | - | Abnormal Hair Growth | 7, 8 | Hom |
| *Tcf7* | Transcription factor 7, T cell specific | - | - | 26642437 | Abnormal Tactile Response | 6, 8, 13, 14, 19 | Hom |
| *Tedc1* | Tubulin epsilon and delta complex 1 | - | - | - | Abnormal Skin Morphology | 6, 13, 14 | Het |
| *Tfec* | Transcription factor EC | - | - | - | Abnormal Hair Pigmentation | 8, 14 | Hom |
| *Timmdc1* | Translocase of inner mitochondrial membrane domain containing 1 | - | - | - | Abnormal Hair Appearance | None | Het |
| *Tiparp* | TCDD-inducible poly polymerase | - | - | 25975270 | Scaly Skin | 1, 6, 12, 13, 16, 18 | Hom/Het |
| *Tmem121b* | Transmembrane protein 121B | - | - | - | Abnormal Skin Morphology | None | Hom |
| *Tmem30b* | Transmembrane protein 30B | - | - | - | Abnormal Hair Pigmentation | 1, 3, 6, 9, 12, 13, 16, 19 | Hom |
| *Tmem68* | Transmembrane protein 68 | - | - | - | Abnormal Tactile Response | 13, 14 | Hom |
| *Tmem74* | Transmembrane protein 74 | - | - | - | Abnormal Skin Morphology | 6, 9 | Hom |
| *Tmem79* | Transmembrane protein 79 | Eczema 24060273 | - | 30463955 | Abnormal Hair Appearance | 6, 13, 14, 15 | Hom |
| *Tmprss6* | Transmembrane serine protease 6 | - | - | 23922777 | Abnormal Skin Morphology | 7, 8 | Hom |
| *Tnni2* | Troponin 1, skeletal, fast 2 | - | - | - | Abnormal Skin Morphology | 14 | Het |
| *Trim39* | Tripartite motif-containing 39 | - | - | - | Abnormal Vibrissae Morphology | 7, 13, 14 | Hom |
| *Tuft1* | Tuftelin 1 | - | - | - | Abnormal Hair Appearance, Abnormal Vibrissae Morphology | 6, 14, 15, 19 | Hom |
| *Twf1* | Twinfilin acting binding protein 1 | - | - | - | Abnormal Skin Morphology | None | Hom |
| *Ube2b* | Ubiquitin-conjugating enzyme E2B | - | - | 26223869 | Abnormal Hair Appearance | 12, 13, 14 | Het |
| *Usp19* | Ubiquitin specific peptidase 19 | - | - | 29901692 | Abnormal Hair Appearance | 6, 14, 15 | Het |
| *Usp29* | Ubiquitin specific peptidase 29 | - | - | - | Abnormal Skin Morphology | 7, 13 | Hom |
| *Usp39* | Ubiquitin specific peptidase 39 | - | - | - | Abnormal Hair Pigmentation | None | Het |
| *Vamp3* | Vesicle-associated membrane protein 3 | - | - | 28931526 | Abnormal Vibrissae Morphology | 6, 7, 14, 18 | Hom |
| *Vps4a* | Vacuolar protein sorting 4A | - | - | - | Abnormal Skin Morphology | 6 | Het |
| *Wdr12* | WD repeat domain 12 | - | - | - | Abnormal Hair Pigmentation | 13 | Het |
| *Wfdc18* | WAP four-disulfide core domain 18 | - | - | - | Abnormal Hair Appearance | None | Hom |
| *Wrnip1* | Werner helicase interacting protein 1 | - | - | - | Abnormal Tactile Response | 6, 13, 14 | Hom |
| *Xk* | X-linked Kx blood group | - | - | 24405768 | Abnormal Skin Morphology | None | Hom |
| *Xxylt1* | Xyloside xylosyltransferase 1 | - | - | - | Abnormal Vibrissae Morphology | 14, 15 | Hom |
| *Xylb* | Xylulokinase homolog | - | - | - | Abnormal Tactile Response | 7, 12, 13, 14, 15 | Hom |
| *Ywhaz* | Tyrosine 3-monooxygenase/tryptophan 5-monooxygenase activation protein, zeta polypeptide | - | Skin Inflammation 29455850 | - | Abnormal Skin Morphology | 6, 7, 8, 14 | Het |
| *Zfp148* | Zinc finger protein 148 | - | - | 23405202 | Abnormal Skin Coloration | 9 | Het |
| *Zfp451* | Zinc finger protein 451 | - | - | - | Abnormal Skin Morphology | 1, 13 | Hom |
| *Zswim5* | Zinc finger SWIM-type containing 5 | - | - | - | Abnormal Hair Pigmentation | None | Hom |
| *Zzef1* | Zinc finger, ZZ-type and EF-hand domain containing 1 | - | - | - | Abnormal Hair Pigmentation | 4, 6, 7, 13, 14, 15 | Hom |

Supplementary Table 2. Knockout mice with oculocutaneous phenotypes. An example of a published mouse and human ocular abnormality associated with each gene is listed if available (with PMID provided), as well as whether one or more (non-KOMP) targeted mutations have been studied for each gene. Ocular phenotypic findings and zygosity of the knockout mice from the screening in the IMPC pipeline as also presented. Among the 307 knockout mice with integument abnormalities, 52 have associated ocular abnormalities. For 17 of these 52 knockout mice, one or more knockout mutations have previously been published as having ocular or integument phenotypes, and 22 have published knockout mice (eight of which did not report ocular or integument abnormalities). Therefore, in total 35 of the 52 knockouts represent potential novel models of oculocutaneous disease.

| Gene | *Gene Name* | *Mouse Eye PMID* | *Human Eye PMID* | *Knock-out Mouse* | *Ocular Phenotype* | Zygosity |
| --- | --- | --- | --- | --- | --- | --- |
| *Ablim1* | Actin-binding LIM protein 1 | Retina Development 12849746 | - | 12849746 | Ocular Hemorrhage | Hom |
| *Acvr2b* | Activin A receptor, type 2B | - | - | - | Abnormal Eye Morphology | Het |
| *Alg10b* | ALG10B, alpha-1, 2-Glucosyltransferase | - | - | - | Abnormal Optic Vesicle Formation | Hom |
| *Anapc15* | Anaphase promoting complex subunit 15 | - | - | - | Microphthalmia | Hom |
| *Ash1l* | ASH1 like histone lysine methyltransferase | - | - | - | Impaired Pupillary Light Reflex | Het |
| *Barx2* | BarH-like homeobox 2 | Ocular Gland Development 21750040 | - | 21750040 | Abnormal Eyelid Aperture, Corneal Opacity | Hom |
| *Bex2* | Brain expressed X-linked 2 | Optic Nerve Stroke  16541015 | - | 25143383 | Abnormal Lens Morphology | Hem |
| *Bms1* | BMS1, ribosome biogenesis factor | - | - | - | Cataract | Het |
| *Carmil2* | Capping protein regulator and myosin 1 linker 2 | - | - | - | Increased Total Retinal Thickness | Hom |
| *Chic2* | Cysteine-rich hydrophobic domain 2 | - | - | - | Abnormal Iris Morphology | Het |
| *Chst8* | Carbohydrate sulfotransferase 8 | - | - | - | Abnormal Eye Morphology | Hom |
| *Clk1* | CDC-like kinase 1 | - | - | 27769915 | Abnormal Retina Morphology | Hom |
| *Cog6* | Component of oligomeric golgi complex 6 | - | - | - | Abnormal Corneal Morphology | Hom |
| *Cotl1* | Coactosin-like 1 (Dictyostelium) | - | - | - | Abnormal Lens Morphology | Hom |
| *Cox6b1* | Cytochrome c oxidase, subunit 6B1 | - | - | - | Microphthalmia | Hom |
| *Dbn1* | Debrin 1 | - | - | - | Decreased Total Retinal Thickness | Hom |
| *Dcp2* | Decapping MRNA 2 | - | - | - | Abnormal vitreous body morphology | Het |
| *Dnase1l2* | Deoxyribonuclease 1-like 2 | - | - | 28743926 | Abnormal Eye Morphology | Hom |
| *Dnm1l* | Dynamin 1-like | - | Dominant Optic Atrophy 28969390 | 22387883 | Persistence of Hyaloid Vasculature | Het |
| *Dsg1b* | Desmoglein 1 beta | - | - | - | Abnormal Eye Morphology | Hom |
| *Dtnbp1* | Dystrobrevin binding protein 1 | - | Hermansky Pudlak Syndrome 12923531 | - | Iris pigmentation, retina morphology | Hom |
| *Fndc3b* | Fibronectin type III domain containing 3B | - | Keratoconus 23291589 | - | Abnormal Eye Morphology | Het |
| *Ggps1* | Geranylgeranyl diphosphate synthase 1 | - | - | - | Cataract | Het |
| *Gp6* | Glycoprotein 6 | - | - | 17991808 | Abnormal Anterior Chamber Depth | Hom |
| *Hdgfl3* | HDGF like 3 | - | - | - | Abnormal Eye Morphology | Hom |
| *Kansl1* | KAT8 regulatory NSL complex subunit 1 | - | Eye Development 26293599 | 28704368 | Abnormal Retinal Outer Nuclear Layer, Decreased Total Retinal Thickness | Het |
| *Kdm8* | Lysine specific demethylase 8 | - | - | - | Abnormal Iris Pigmentation | Het |
| *Klhdc2* | Kelch domain containing 2 | - | - | - | Corneal Opacity | Het |
| *Lyst* | Lysosomal trafficking regulator | Chediak-Higashi Syndrome 8717042 | Chediak-Higashi Syndrome 8717042 | 19029039 | Abnormal iris and retina morphology and pigmentation | Hom |
| *Mfsd8* | Major facilitator superfamily domain containing 8 | Photoreceptor Degeneration 24423645 | Retinal Dystrophy 28586915 | 24423645 | Impaired pupillary reflex, abnormal retina morphology | Hom |
| *Mogs* | Mannosyl-oligosaccharide glucosidase | - | - | - | Anophthalmia | Hom |
| *Myo10* | Myosin X | Hyaloid Vascular Regression 29229982 | - | 30679680 | Persistence of hyaloid vasculature | Hom |
| *Mysm1* | Myb like, SWIRM and MPN domains 1 | - | - | 26915790 | Narrow eye opening | Hom |
| *Nadk2* | NAD kinase 2, mitochondrial | - | - | 28923496 | Persistence of hyaloid vasculature | Het |
| *Nck1* | Non-catalytic region of tyrosine kinase adaptor protein 1 | Ischemic Neuropathy 30150707 | - | 25398386 | Abnormal Eye Morphology | Hom |
| *Nfkb1* | Nuclear factor of kappa light polypeptide gene enhancer in B cells 1 | - | - | 28726772 | Abnormal Corneal Morphology | Hom |
| *Plac8* | Placenta-specific 8 | - | - | 30671116 | Cataract | Hom |
| *Prdm5* | PR domain containing 5 | - | Brittle Cornea Syndrome 27032025 | - | Abnormal Optic Nerve Morphology, Abnormal Retinal Vasculature | Hom |
| *Rcc2* | Regulator of chromosome condensation 2 | - | - | - | Abnormal Optic Nerve Morphology | Hom |
| *Rspo1* | R-spondin 1 | Globe Axial Length  24144296 | Corneal Endothelial Proliferation 25277232 | 21484214 | Abnormal Anterior Chamber Depth, Abnormal Vitreous Morphology | Hom |
| *Sdc2* | Syndecan 2 | Corneal Healing 28973369 | - | 23233348 | Abnormal Lens Morphology | Hom |
| *Sdr42e1* | Short chain dehydrogenase/reductase family 42E, member 1 | - | - | - | Abnormal Cornea Morphology | Hom |
| *Sema3f* | Sema domain, immunoglobulin domain, short basic domain, secreted 3F | Retinal Development 28373097 | - | 20006658 | Cataract | Hom |
| *Sgip1* | SH3-domain GRB2-like interacting protein 1 | - | - | - | Abnormal Lens Morphology | Het |
| *Sik3* | SIK family kinase 3 | - | - | 22318228 | Abnormal Corneal Morphology | Hom |
| *Slc17a9* | Solute carrier family 17 member 9 | - | - | - | Abnormal Eye Morphology | Hom |
| *Slc24a5* | Solute carrier family 24 member 5 | Oculocuteaneous Albinism 18424845 | Oculocuteanous Albinism 23364476 | 18424845 | Abnormal Iris Transillumination, Abnormal Retina Morphology | Hom |
| *Slc38a10* | Solute carrier family 38 member 10 | - | - | - | Abnormal Retinal Morphology | Hom |
| *Sparc* | Secreted protein acidic and cysteine rich | Anterior Segment Biometry 28122087 | Glaucoma 23599341 | 21384171 | Abnormal Lens Morphology | Hom |
| *Tcf7* | Transcription factor 7, T cell specific | - | - | - | Cataract | Hom |
| *Tmem30b* | Transmembrane protein 30B | - | - | - | Abnormal Eye Morphology | Hom |
| *Tuft1* | Tuftelin 1 | - | - | - | Abnormal Eyelid Morphology | Hom |
